# An information network flow approach for measuring functional connectivity and predicting behavior

**DOI:** 10.1002/brb3.1346

**Published:** 2019-07-09

**Authors:** Sreejan Kumar, Kwangsun Yoo, Monica D. Rosenberg, Dustin Scheinost, R. Todd Constable, Sheng Zhang, Chiang‐Shan R. Li, Marvin M. Chun

**Affiliations:** ^1^ Department of Psychology Yale University New Haven Connecticut; ^2^ Department of Psychology University of Chicago Chicago Illinois; ^3^ Department of Radiology and Biomedical Imaging Yale School of Medicine New Haven Connecticut; ^4^ Interdepartmental Neuroscience Program Yale University New Haven Connecticut; ^5^ Department of Neurosurgery Yale School of Medicine New Haven Connecticut; ^6^ Department of Psychiatry Yale School of Medicine New Haven Connecticut; ^7^ Department of Neuroscience Yale School of Medicine New Haven Connecticut

**Keywords:** functional connectivity, information flow, predictive model, resting‐state fMRI connectivity, sustained attention

## Abstract

**Introduction:**

Connectome‐based predictive modeling (CPM) is a recently developed machine‐learning‐based framework to predict individual differences in behavior from functional brain connectivity (FC). In these models, FC was operationalized as Pearson's correlation between brain regions’ fMRI time courses. However, Pearson's correlation is limited since it only captures linear relationships. We developed a more generalized metric of FC based on information flow. This measure represents FC by abstracting the brain as a flow network of nodes that send bits of information to each other, where bits are quantified through an information theory statistic called transfer entropy.

**Methods:**

With a sample of individuals performing a sustained attention task and resting during functional magnetic resonance imaging (fMRI) (*n* = 25), we use the CPM framework to build machine‐learning models that predict attention from FC patterns measured with information flow. Models trained on *n *− 1 participants’ task‐based patterns were applied to an unseen individual's resting‐state pattern to predict task performance. For further validation, we applied our model to two independent datasets that included resting‐state fMRI data and a measure of attention (Attention Network Task performance [*n* = 41] and stop‐signal task performance [*n* = 72]).

**Results:**

Our model significantly predicted individual differences in attention task performance across three different datasets.

**Conclusions:**

Information flow may be a useful complement to Pearson's correlation as a measure of FC because of its advantages for nonlinear analysis and network structure characterization.

## 1 INTRODUCTION

The brain's functional organization at rest and during task engagement can be studied with functional connectivity analyses of fMRI signals. Functional connectivity between two regions of the brain, or the statistical association between their activation over time, is frequently measured as Pearson's correlation between their respective fMRI signals (Heuvel & Pol, 2010; Smith et al., 2013). Whole‐brain functional connectivity can be represented as a matrix of the pairwise correlations across every region (Smith et al., 2013). This matrix, or “functional connectome,” is often interpreted as a fully connected network in which the nodes are brain regions and edges between them have a weight equal to the respective correlation (Smith et al., 2013).

Although functional connectivity measured with Pearson's correlation has provided valuable insights into features of large‐scale brain organization common to the healthy population (van Dijk, Sabuncu, & Buckner, 2012; Fox et al., 2005; Power et al., 2011; Yeo et al., 2011) and unique across individuals (Finn et al., 2015; Rosenberg, Finn, et al., 2016a), two theoretical disadvantages of Pearson's correlation may limit its utility as a measure of functional brain organization. First, Pearson's correlation does not account for the possibility of nonlinear relationships between two regions’ signals. Previous research has shown that nonlinear analysis of fMRI signals can reveal results that linear analysis cannot. For example, Su, Wang, Shen, Feng, and Hu (2013) found that functional connectivity calculated from nonlinear analysis differentiated schizophrenic patients from healthy patients with higher accuracy than linear functional connectivity. They also found that the strength of certain nonlinear functional connections increased in patients with schizophrenia, whereas their linear counterparts did not. Second, Pearson's functional connectome only contains pairwise interactions in the brain, but does not contain explicit information on how these connections are organized. Investigating the topological structure of these connections can give some insight into how these connections are organized as a part of a more complex network (Bassett & Bullmore, 2006; Heuvel & Pol 2010; Smith et al., 2013).

In this paper, we propose a new measure of functional connectivity that addresses these two disadvantages by unifying both nonlinear analysis of pairwise fMRI signals and large‐scale network analysis. The goal of this study was to validate this new measure of functional connectivity—information flow measured as the maximum flow between nodes whose capacities are defined with transfer entropy—by demonstrating that it predicts individual differences in behavior. To do this, utilizing an approach similar to an approach by Yoo et al. (2018), we test whether models based on information flow, in comparison with models based on the Pearson correlations, can generalize to predict attention performance across three completely independent datasets (Jangraw et al., 2018; Rosenberg, Finn, et al., 2016a; Rosenberg, Hsu, Scheinost, Constable, & Chun, 2018; Rosenberg, Zhang, et al., 2016b; Rosenberg et al., 2018; Yoo et al., 2018). Datasets include fMRI data collected from healthy adult participants who performed a gradual‐onset continuous performance task, Attention Network Task (ANT), or stop‐signal task during fMRI. Although there has been research in investigating functional connectivity in the brain using information theory (Dimitrov, Lazar, & Victor, 2011; Garofalo, Nieus, Massobrio, & Martinoia, 2009; Mäki‐Marttunen, Diez, Cortes, Chialvo, & Villarreal, 2013; Vergara, Miller, & Calhoun, 2017; Vicente, Wibral, Lindner, & Pipa, 2010; Viol, Palhano‐Fontes, Onias, Araujo, & Viswanathan, 2017), this is the first research that, to our knowledge, unifies information‐theoretic analysis with graph theory to predict human behavior from fMRI data. Significant predictions would show that information flow may be a useful alternative to Pearson's correlation as a measure of functional connectivity to predict behavior due to its theoretical advantages in addressing both nonlinear analysis and characterizing network structure as well as success in predicting individual differences in behavior.

## 2 METHODS

### 2.1 Information flow as a measure of functional connectivity

#### 2.1.1 Background

Here, we motivate our new measure of functional connectivity through an abstraction that involves using information theory to represent the brain as a flow network of information bits. It may be particularly useful to explore functional brain connectivity through information theory because the brain is an information processing system (Reinagel, 2000). Transfer entropy (TE) is an information‐theoretic metric that can measure both linear and nonlinear information transfer between two systems (Schreiber, 2000). The TE from signal *A* to signal *B* answers the question, “How much information does the past state of *A* contain about the future state of *B*, given that we know the past state of *B*?” (Wibral, Vicente, & Lindner, 2014). Unlike Pearson's correlation, TE is a directed metric, meaning that the TE from *A* to *B* is different from the TE to *B* to *A*. For example, Schreiber (2000) used TE on heart and breath rate data and found that the TE from heart rate to breath rate was greater than the TE from breath rate to heart rate.

Instead of coding the functional connectivity between two regions as the correlation of their respective time series, we can use TE to code their relationship as the number of bits transferred from one to the other. Transfer entropy has previously been used in a variety of neuroimaging studies for functional connectivity analysis (Wibral et al., 2014). For example, Mäki‐Marttunen and colleagues used TE to analyze resting‐state functional connectivity in comatose patients as compared to control subjects, and found that the TE calculated from left intrahemispheric ROIs could be a potential marker for large‐scale disturbance of brain function in these patients (Mäki‐Marttunen et al., 2013). Although other measures can characterize nonlinear interactions between signals, here we focus on a metric from information theory given that we were motivated by abstracting the brain as a flow network of information bits. We chose TE rather than alternative information theory metrics such as mutual information because it is a directed measure (whereas mutual information is an undirected measure) and flow networks are most frequently analyzed as directed graphs (Ahuja, Magnanti, & Orlin, 2014).

It is important to emphasize that TE is *predictive* information transfer, which is distinct from *causal* interaction (Lizier & Prokopenko, 2010). To determine causal interaction, intervention in the system is necessary. An information‐theoretic approach to information transfer like TE is more concerned with how knowing one process affects one's ability to predict the other and relies on studying systems in the absence of intervention. For our purposes, we are interested in information transfer in a purely computational sense, and do not use this measure to infer causal relationships between activity in distinct brain regions. Furthermore, predictive information transfer is a statistical measure that does not characterize physical pathways. Thus, we refer to information transfer as representing abstract, functional pathways rather than physical brain connections.

The TE from region *A* to region *C* measures the information transfer from *A* to *B* given their respective time series. To fully characterize the functional relationship between *A* and *C*, however, we may wish to consider other nodes’ time series that can make indirect functional connections, or alternate paths, between them. Previous research has shown that, although direct structural connections alone do not predict functional connectivity well, incorporating indirect structural paths can improve predictions of functional connectivity (Deligianni et al., 2011; Røge et al., 2017). It is possible that, analogously, predictions of behavior can be improved by taking into account nodes on indirect functional paths along with direct functional paths. In graph theory, two nodes can be connected both by a direct connection, or edge, and indirect pathways that go through alternate nodes. For example, let us say node *A* passes information to node *C* through node *B*, but node *B* might modify that information. Taking into account node *A*’s interaction with node *B*, node *B*’s interaction with node *C*, along with node *A*’s direct interaction with node *C*, would give us stronger evidence that node *A* is sending node *C* a message. This is different from the standard approach of partial correlation, where we take into account node *C*’s activity in order to isolate the relationship between nodes *A* and *B*. With our approach, we are not trying to use other nodes’ activity to isolate the relationship between two nodes, but trying to build a feature of the interaction of those two nodes that takes into account the underlying network structure. In general, taking into account the activity of intermediate nodes on these alternative pathways between two nodes will allow us to incorporate both information about information transfer measured between the two nodes’ time series and how those two nodes are connected in the context of the larger network structure.

**Figure 1:**
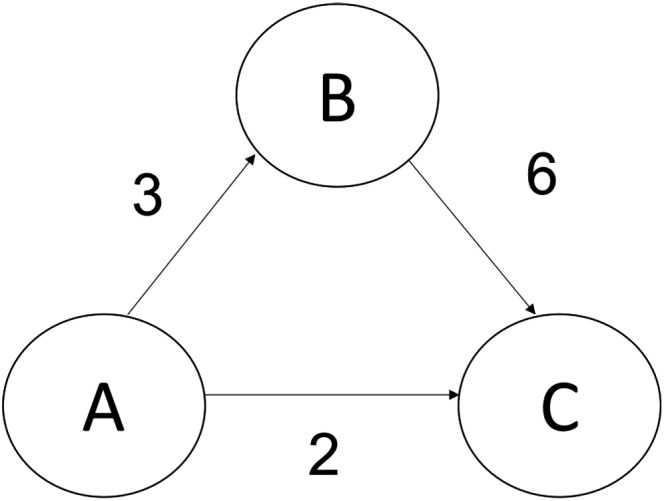
Simple example of the maximum flow problem: a directed graph where each edge's weight represents the edge's capacity, which is the greatest amount of flow that can go through that edge. Although one can only flow two units directly from A to C, one can also flow three units using the alternative path from A to B and then from B to C, which means the maximum flow from A to C is equal to 5

To add consideration of nodes on indirect functional paths between pairs of brain regions, we use the concept of maximum flow (Ahuja et al., 2014), a well‐known problem in optimization theory. Maximum flow problems start with directed weighted graphs, where each edge's weight represents the largest number of units one can transport through that particular edge (also known as capacity). The maximum flow from a source node to a sink node is the maximum amount one can “flow” from the source to the sink given these capacities on transportation. The amount one can flow does not only depend on the weight (capacity), but also the specific underlying structure of the edges in the graph. In the example shown in Figure 1, even though the capacity of the edge between *A* and *C* is 2, the maximum flow between *A* and *C* is 5, because there is an alternate path that goes from *A* to *B* to *C*. Maximum flow provides a good way to characterize how two nodes are connected with each other using both their direct edge (*A*‐>*C*) and indirect pathways (*A*‐>*B*‐>*C*).

Maximum flow has previously been used to characterize alternative *structural* connectivity. Yoo et al. (2015) characterize structural connectivity between regions as the maximum flow between the regions’ corresponding nodes on a network calculated from MRI data. Here, we employ maximum flow to characterize *functional* connectivity. We quantify functional connectivity between regions as the greatest number of bits one region can flow to another region, where direct connections between regions have a capacity equal to their TE. Since we represent the capacities as transfer entropies, each capacity represents the greatest number of bits that can transfer within that edge. We are using maximum flow as a way to abstract the brain as a flow network of nodes that send bits of information to each other, where the bits through specific edges are quantified through TE. The addition of maximum flow gives our measure graph‐theoretic properties. That is, by defining functional connectivity with maximum flow, we are taking into account how those nodes are connected within the context of the larger underlying network of the brain.

#### 2.1.2 Proposed connectivity measure

##### Overview of connectivity matrix construction

**Figure 2:**
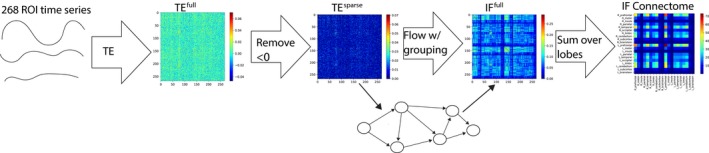
Steps used to construct an individual's information flow connectivity matrix. (a) Each individual's fMRI data are parcellated into *n* ROI time series, depending on the parcellation used (see *Step 1: parcellation*). (b) A full transfer entropy matrix (TE^full^) is populated with all pairwise transfer entropies among the ROI time series. (c) Negative transfer entropy values are set to 0 to create TE^sparse^ (see *Step 2: measuring functional connectivity with transfer entropy*). (d) Cells in the TE^sparse^ matrix are used to construct graphs in which edges are defined as the maximum flow between each pair of nodes. (e) These graphs are represented as a new maximum flow matrix (see *Step 3: computing higher‐order connectivity features with maximum flow*). (f) The maximum flow matrix is then reduced over the anatomical lobe groups (see *Step 3: computing higher‐order connectivity features with maximum flow*)

Figure 2 shows the overall steps we use to construct the information flow connectivity matrices and describe in the following sections.

##### Step 1: parcellation

Preprocessed data were used to calculate a task‐based and a resting‐state functional connectivity matrix for each participant in the gradCPT dataset, and a resting‐state functional connectivity matrix for each participant in the external datasets. We defined nodes were using the Shen 268‐node functional brain atlas, which includes the cortex, subcortex, and the cerebellum (Shen, Tokoglu, Papademetris, & Constable, 2013). The Shen atlas was warped from MNI (Montreal Neurological Institute)  space into single‐subject space using linear and nonlinear registrations between the functional images, anatomical scans, and MNI brain. For every node, a mean task‐based time course was calculated by averaging the time courses of all of its constituent voxels during task performance, and a mean resting‐state time course was calculated by averaging these time courses during rest. Although here we used the parcellation from Shen et al. (2013), our measure can be applied with any other type of parcellation.

##### Step 2: measuring functional connectivity with TE

Transfer entropy serves as the initial connectivity metric to calculate higher‐order connectivity features. Transfer entropy is an information‐theoretic metric that measures information transfer between two systems (Schreiber, 2000). The transfer entropy from process *A* to *C* tells how much information does the past state of *A* (*A_n_*) contain about the future state of *C* (*C_n_*_ + 1_) given that we know the past state of *C* (*C_n_*). This idea is quantified as follows:

$${\text{TE}}_{A\to C}=\sum p\left(C_{n+1},C_{n}^{\left(k\right)},A_{n}^{\left(l\right)}\right)\text{log}\frac{p\left(C_{n+1}|C_{n}^{\left(k\right)},A_{n}^{\left(l\right)}\right)}{p\left(C_{n+1}|C_{n}^{\left(k\right)}\right)}$$

Briefly, this equation uses the concept of entropy and conditional probability distributions to quantify the “incorrectness” of the Markov property: $p\left(C_{n+1}|C_{n}^{\left(k\right)},A_{n}^{\left(l\right)}\right)=p\left(C_{n+1}|C_{n}^{\left(k\right)}\right)$. If this property holds, our ability to predict the next state of *C* using both the previous states of *A* and *C* is the same as our ability to predict the next state of *C* using just the previous state of *C*. This property would mean that there is no information transfer from *A* to *C* (Schreiber, 2000).

Multiple algorithms have been developed and used to estimate TE for continuous data. Here, we use the Kraskov, Stogbauer, and Grassberger (KSG) (2018) technique which estimates a probability density function for a time series using Kernel estimation and alters the kernel width to adjust to the data using a nearest neighbor calculation (Lizier, 2014). The KSG approach is often used as the “best of breed solution” for TE estimation (Lizier, 2014). We used the open‐source Java Information Dynamics Toolkit to calculate the transfer entropies (Lizier, 2014).

We calculated a full 268 x 268 “TE Matrix” (Figure 2b) for each individual, where each cell *M_ij_* contains the estimated TE from node *i* to node *j*. In a graph theory context, each TE matrix represents an adjacency matrix of individual brain networks. Since TE is a directed metric, this graph is a directed graph (the edge from node *A* to *C* is different from the edge from node *C* to *A*). Since the TE matrix is a full matrix, this would also be a complete graph (there exist a forward edge and a back edge for every two nodes).

Because the KSG algorithm is an estimator with a variance associated with it (Lizier, 2014), the estimated TE can be negative if the true TE between the processes is (or very close to) 0. Transfer entropy cannot be theoretically negative (Schreiber, 2000), so a measured negative TE would be a measurement error. Therefore, after calculating the full TE matrix, we find the sparse 268 × 268 TE Matrix (Figure 2c) by removing transfer entropies that were measured as negative (setting them to 0).

##### Step 3: computing higher‐order connectivity features with maximum flow

The next step in calculating our measure is to add information about the underlying network topology to the estimation of functional connectivity between nodes *A and C*. To do this, we used maximum flow to construct higher‐order connectivity features that include nodes on alternative paths of information flow.

The maximum flow problem is as follows: Given a network, what is the greatest amount of units one can flow from a source node to a sink node using the network's edges if each edge's weight represents the capacity of how many units can flow through that edge. By defining connectivity with maximum flow, calculation of connectivity between two nodes also takes into account how those nodes are connected to other nodes. For all maximum flow calculations, we used the implementation in Python's NetworkX Package (Hagberg, Daniel, & Pieter, 2008).

After calculating the sparse TE matrix for each individual (see previous section), we then construct the full 268 × 268 Information Flow (IF^full^) Matrix (Figure 2e) using the sparse TE matrix, where IF^full^_ij_ contains the maximum information flow from node *i* to node *j* which was calculated using a network defined by the sparse TE matrix (Figure 2d). Specifically, we use the sparse TE matrix as the adjacency matrix for the directed graph used for maximum flow.

We calculated three different versions of the information flow matrices: full information flow matrix with no restriction, full information flow matrix with anatomical restriction, and reduced information flow matrix. The full information flow matrix was calculated by using the entire sparse TE matrix to calculate maximum flow for each pair of nodes. The other two matrices are calculated using a similar process, but with the introduction of an anatomical restriction.

The full information flow matrix with anatomical restriction calculates information flow among nodes of the same macroscale brain region. To do this, we impose a restriction on the edges we consider when calculating maximum flow. Nodes were grouped in the 10 brain lobes in each hemisphere: prefrontal, motor, insula, parietal, temporal, occipital, limbic, cerebellum, subcortical, and brainstem. This is the same lobe scheme described in Finn et al. (2015).

Let us say lobe(*A*) represents the anatomical lobe that node *A* resides in, lobe(*C*) represents the anatomical lobe that node *C* resides in, and Maxflow(*A*, *C*, *E*) represents the maximum flow from node *A* to node *C* on a network with the set of edges *E*. TE^sparse^ represents the sparse TE matrix.

$${\text{IF}}_{\mathrm{AC}}^{\text{full}}=\text{Maxflow(}A,C,E_{\mathrm{AC}})$$

$$E_{\mathrm{AC}}=\left\{\left(i,j\right)\in {\text{TE}}^{\text{sparse}}\forall \;\text{node}i\in \text{lobe(}A\right),\forall \;\text{node}j\in \text{lobe}\left(C\right)\}$$

Note that if nodes *A* and *C* reside in the same lobe, then the edge set will be all edges that reside inside that lobe. Also, note that the number of edges in the set *E_AC_* can vary since they come from the sparse TE matrix, which does not necessarily have an edge for every two nodes.

The reduced form information flow matrix (IF; Figure 2f) is a 20 × 20 matrix where IF*_xy_* is the total information flow going from lobe *x* to lobe *y* in bits, where paths of information are restricted using the procedure described in the previous paragraph. This is calculating by summing individual flows in IF^full^:

$${\text{IF}}_{\mathrm{xy}}=\sum_{\begin{array}{c} \text{node}\;i\in x\\ \text{node}\;j\in y \end{array}}{\text{IF}}_{\mathrm{ij}}^{\text{full}}$$

If *x* and *y* are distinct lobes, then the cell IF*_xy_* measures the summed information flow from lobe *x* to lobe *y*. Note that this is a directed measure (flow from *x* to *y* is different than flow from *y* to *x*), since the maximum flow of a graph is a directed quantity. If *x* and *y* are the same lobe, then the cell IF*_xy_* will contain the summed information flow within that lobe. This step is used to reduce the amount of total features for each individual from 71,824 in the full matrix to 400 in the reduced matrix. This reduction in the dimensionality of the feature space helps avoid overfitting predictive models.

### 2.2 Using information flow to predict individual differences in behavior

#### 2.2.1 Data description

##### Internal validation: gradCPT dataset

Predictive models were defined and internally validated with leave‐one‐subject‐out cross‐validation using a dataset described in detail in previous work (Rosenberg, Finn, et al., 2016a). Briefly, this sample included 25 healthy adult participants performing the gradual‐onset continuous performance task (gradCPT; (Esterman, Noonan, Rosenberg, & Degutis, 2012; Rosenberg, Noonan, Degutis, & Esterman, 2013)), a test of sustained attention, and resting during functional magnetic resonance imaging (fMRI). GradCPT performance was assessed with sensitivity (*d′*). This measure was used to assess sustained attention during the gradCPT task in previous work (Rosenberg, Finn, et al., 2016a; Yoo et al., 2018) and was found to have very high reliability (Rosenberg, Finn, et al., 2016a). This measure was also confirmed to not be related to head motion in this sample (Rosenberg, Finn, et al., 2016a).

Scan sessions included a high‐resolution anatomical scan (MPRAGE), a 2D T1‐weighted image with the same slice prescription as the functional images for registration purposes, a 6‐min resting‐state run, three 13:44‐min gradCPT runs, and a second 6‐min rest run. Each gradCPT run included 8 s of fixation (excluded from analysis) followed by three 4‐min blocks of the task interleaved with 32‐s breaks. Volumes collected during break periods were also excluded from analysis.

Functional runs included 824 (task) or 363 (rest) whole‐brain volumes acquired using a multiband echo‐planar imaging (EPI) sequence. Parameters were as follows: repetition time (TR) = 1,000 ms, echo time (TE) = 30 ms, flip angle = 62°, acquisition matrix = 84 × 84, in‐plane resolution = 2.5 mm^2^, 51 axial‐oblique slices parallel to the ac‐pc line, slice thickness = 2.5, multiband 3, and acceleration factor = 2. MPRAGE parameters were as follows: TR = 2,530 ms, TE = 3.32, flip angle = 7°, acquisition matrix = 256 × 256, in‐plane resolution = 1.0 mm^2^, slice thickness = 1.0 mm, and 176 sagittal slices. A 2D T1‐weighted image with the same slice prescription as the functional images was also collected to aid with registration.

Image preprocessing was performed using BioImage Suite (Joshi et al., 2011) and custom MATLAB scripts. SPM8 (http://www.fil.ion.ucl.ac.uk/spm/software/spm8/) was used to perform motion correction. The following were regressed from the data: mean signal from cerebrospinal fluid, white matter, linear and quadratic drift, and gray matter and a 24‐parameter motion model (six motion parameters, six temporal derivatives, and their squares). Finally, data were temporally smoothed with a zero mean unit variance Gaussian filter.

Due to excessive head motion (>2 mm translation, >3° rotation, or 0.15 mm mean frame‐to‐frame displacement), one resting run from two participants and one task run from five participants were excluded from analysis. Head motion, calculated as mean frame‐to‐frame displacement, did not correlate with d′ in any of the three task runs. Additional details are provided in Rosenberg, Finn, et al., 2016a.

##### External validation 1: ANT dataset

The predictive model defined in the gradCPT dataset was applied unchanged to three completely independent samples to assess generalizability. The first external validation sample included fMRI data collected as 44 participants performed the ANT (Fan, Mccandliss, Fossellia, Flombaum, & Posner, 2005) and rested (Rosenberg et al., 2018). Three participants were excluded prior to analysis because they had previously participated in the gradCPT study. ANT performance was measured using variability of correct‐trial response times (i.e., RT standard deviation divided by mean), a more sensitive measure of overall attention to the task than accuracy (Rosenberg et al., 2018).

Functional and structural MRI scans were acquired as was done with the gradCPT dataset. Experimental sessions began with a high‐resolution anatomical scan, followed by two 6‐min resting scans and six 7:05‐min task runs. Resting‐state runs included 360 whole‐brain volumes, and task runs included 425 volumes. fMRI data were preprocessed with the same steps as in the gradCPT dataset. Excluded from the analysis were runs with excessive head motion, defined a priori as >2‐mm translation, >3° rotation, or 0.15‐mm mean frame‐to‐frame displacement (Rosenberg, Finn, et al., 2016a; Rosenberg, Zhang, et al., 2016b). For excessive motion, two task runs were excluded from one participant and one task run was excluded from three participants, and. Additional details are provided in Rosenberg et al., 2018.

Because of moderate correlations between head motion and RT variability, we also excluded edges that were correlated with head motion (maximum displacement, maximum rotation, or mean frame‐to‐frame displacement) at the *p *< 0.05 significance level (Rosenberg et al., 2018).

##### External validation 2: stop‐signal task dataset

The data described in Rosenberg, Zhang, et al. (2016b) were used for the second external validation dataset. This sample contained 72 healthy adults that performed a stop‐signal task (four 9:50‐min runs) and rested (one 9:50‐min run) during fMRI scanning. Approximately 40 min before scanning, 24 of these participants were given a single dose of methylphenidate (MPH), a common treatment for attention deficit hyperactivity disorder (ADHD). Resting‐state data were available for 16 participants in the methylphenidate group and 56 participants in the control group. For this paper, we only used resting‐state fMRI data on the external datasets. This dataset was originally described in Farr et al. (2014a, 2014b), and the criteria for excluding subjects are detailed in Rosenberg, Zhang, et al. (2016b). We refer to this dataset as the MPH dataset.

The preprocessing steps were identical to those described above. Runs were excluded for excessive head motion, defined a priori as >2‐mm translation,>3° rotation, or >0.15‐mm mean frame‐to‐frame displacement (Rosenberg, Zhang, et al., 2016b). We used go response rate to measure attention, since it was found to be the response variable most closely related to sustained attention (Rosenberg, Zhang, et al., 2016b).

#### 2.2.2 Connectome‐based predictive modeling

**Figure 3:**
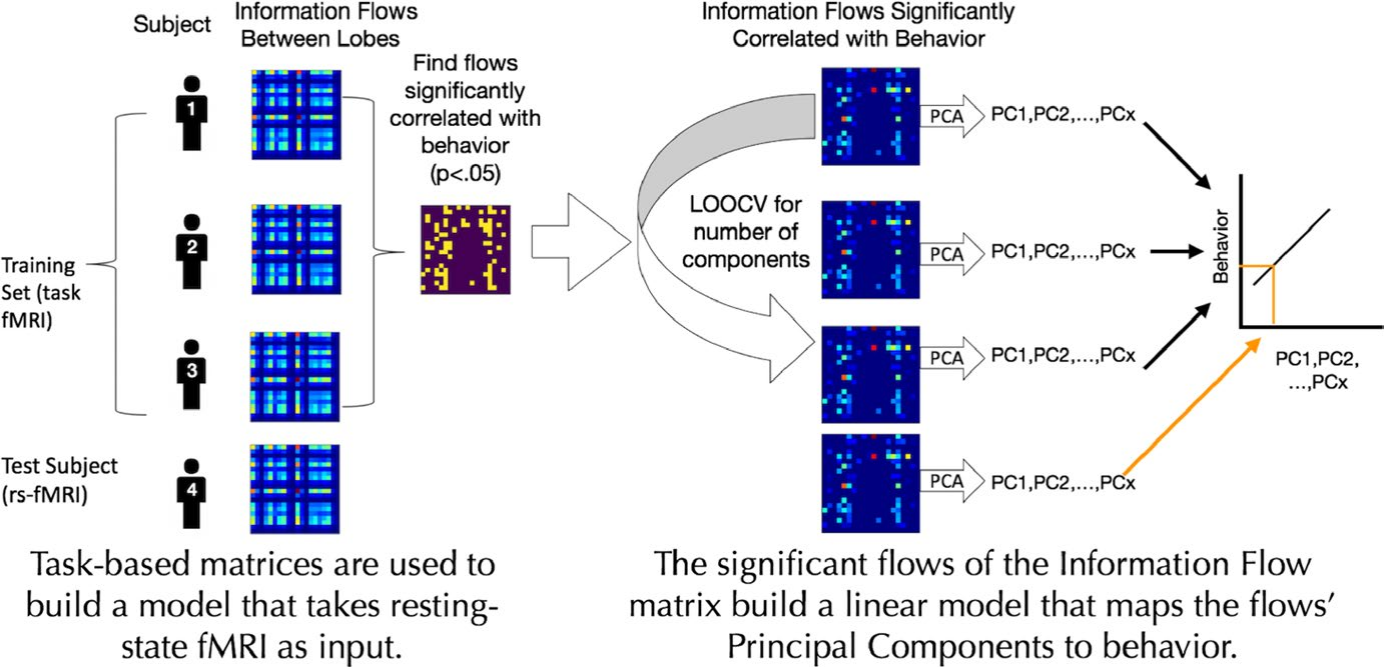
Overview of our predictive modeling pipeline. Task‐based information flow matrices are used to train a linear model that can predict a subject's behavior score using his/her resting‐state information flow connectome

Recent work has demonstrated that individual differences in functional brain organization are related to individual differences in traits and behavior. Thus, if information flow (i.e., the maximum flow between two nodes whose capacities are defined with TE) accurately captures the functional architecture of the brain, it should be able to significantly predict an individual's cognitive tendencies and behavioral performance. Connectome‐based predictive modeling (CPM), a recently developed machine‐learning‐based framework for predicting individual differences in behavior (Shen et al., 2017), has been used, for example, to show that functional connectivity observed during task engagement and rest predicts individual differences in attention (Rosenberg, Finn, et al., 2016a), and fluid intelligence (Finn et al., 2015; Shen et al., 2017). It has also been used to measure various measures of attention such as ADHD symptom severity (Rosenberg, Finn, et al., 2016a), stop‐signal task performance (Rosenberg, Zhang, et al., 2016b), and ANT performance (Rosenberg et al., 2018). We used CPM to generate models of attention using the 20 × 20 information flow matrices from the 25 participants in our training (internal validation) set. The final trained model is a regression model that can be used to predict behavior from resting‐state functional connectivity measured with information flow. Figure 3 shows an overview of our predictive modeling pipeline.

First, flows (individual cells in the information flow, or IF, matrices) that are relevant to behavior are identified by calculating the Spearman rank correlation between each flow in the task information flow matrix across subjects in the training set and the corresponding behavioral scores. Any flows that are not significantly correlated (*p *> 0.05) are taken out of consideration.

The next step is feature aggregation through principal components analysis (PCA) on the selected flows. PCA makes components, linear combinations of the features that maximize variance. When performing PCA, one needs to decide the number of components to use. The number of components is selected with a leave‐one‐subject‐out cross‐validation (LOOCV) loop within the training set. All possible components numbers, from 1 to the number of subjects, are tested within the LOOCV loop, and the number of components that gives the highest prediction performance is selected. Then, the final PCA transformation is applied on the selected flows, and the resulting features are incorporated into a linear regression where the dependent variable is the behavior score.

#### 2.2.3 Model validation

##### Model validation: internal dataset

The gradCPT dataset was used for internal validation (Rosenberg, Finn, et al., 2016a; Rosenberg, Zhang, et al., 2016b). Two information flow matrices are calculated for each subject: one on task and one on resting‐state data. Then, LOOCV is employed to evaluate the model performance. In each iteration, the subjects are separated into a training set (*n* = 24) and a testing set (*n* = 1). Within the training set, task flows (cells on the task IF) relevant to behavior are isolated by performing Spearman's (rank) correlation on each flow with the behavior score, *d′*. Flows that are not significantly correlated with behavior at a threshold of *p* = 0.05 are left out of consideration. Then, a PCA transformation is fitted on the remaining flows. As described in the previous section “Connectome‐based Predictive Modeling,” the number of PCA components is selected based on a nested LOOCV loop inside the 24‐participant training set. In other words, the number of components is set to the number that gives the best performance in a LOOCV loop within the training set. In internal validation, this nested LOO loop is run within the current training set for each iteration of the original LOO loop. The calculated PCA transformation fitted on the training set is then applied on the training set. Then, the same edges selected on the training set are selected on the left‐out subject, and the same PCA transformation calculated on the training set is applied on the left‐out subject. The resulting features on the training set are incorporated into a linear regression to predict the left‐out subject's behavior score.

In order to facilitate interpretability, we then converted each left‐out subjects’ predicted behavior scores into a standardized *z*‐score. The mean and standard deviation used for this *z*‐score is derived from the population of predicted behavior scores predicted from the nested LOOCV left‐out participants’ resting‐state fMRI that we predicted in the previous step. The final score of the novel subject is a *z*‐score that represents how the novel subject's behavior deviates from the predicted behavior of the individuals in the training set, where each individual prediction in the training set was calculated using a nested LOOCV loop.

After each participant has been left out once, predictions are evaluated through Spearman's rank correlation with the actual scores. Although we normalized predicted but not observed behavioral scores, this does not affect the results since we are using Spearman's rank correlation, which only considers the relative ordering of behavior and predicted behavior across individuals. Although there exist alternative model evaluation metrics such as mean squared error, correlation is well suited for evaluating CPM models whose predictions should be considered relative rather than absolute (Rosenberg, Finn, et al., 2016a; Shen et al., 2017).

##### Model validation: external datasets

For external validation, the gradCPT dataset was used as the training data and the ANT and stop‐signal task dataset were used for external validations. The model is built with the gradCPT dataset in the same fashion the model was built in the training set of a single LOOCV iteration in internal validation. The same task‐relevant edges selected within the training set (gradCPT) are used in the testing set, and the same PCA transformation calculated in the training set is applied on the testing set. In other words, the same trained model used in internal validation is used on the external datasets, and the external datasets are completely independent. Again, the number of PCA components is determined using a LOOCV loop within the training set (gradCPT).

Just as in internal validation, we convert each predicted behavior score to a standardized *z*‐score. The mean and standard deviation used for this *z*‐score is derived from the population of predicted behavior scores predicted from the training set (gradCPT) participants’ resting‐state fMRI that we predicted in the previous step with a LOOCV loop. The final score of each subject in the testing set is a *z*‐score that represents how that testing set subject's behavior deviates from the predicted behavior of the individuals in the training set, where each individual prediction in the training set was calculated using a LOOCV loop.

## 3 RESULTS

### 3.1 Information flow connectivity matrices

**Figure 4:**
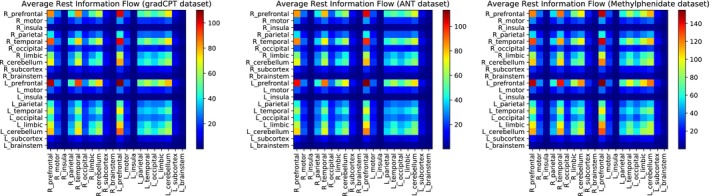
Group averaged resting‐state information flow connectivity matrices across datasets. The scale of the color bar is in bits

Figure 4 shows group averaged resting‐state full information flow matrices across the three datasets. In each of the three datasets, the five highest average flows were within the left prefrontal cortex, from the left prefrontal cortex to the right prefrontal cortex, from the right prefrontal cortex to the left prefrontal cortex, from the right temporal lobe to the left prefrontal cortex, and from the left prefrontal cortex to the right temporal lobe. We performed follow‐up analyses to determine if information flow is correlated with Signal‐to‐Noise ratio (SNR) (see Figure S2 and S3 for further details). We concluded that information flow is not significantly correlated with SNR. 

### 3.2 Internal validation results

Model performance was evaluated using Spearman's rank correlation on predicted scores (each calculated as a left‐out subject within the LOOCV loop) and actual behavior scores. Statistical significance was evaluated through a 10,000‐iteration permutation test.

**Figure 5:**
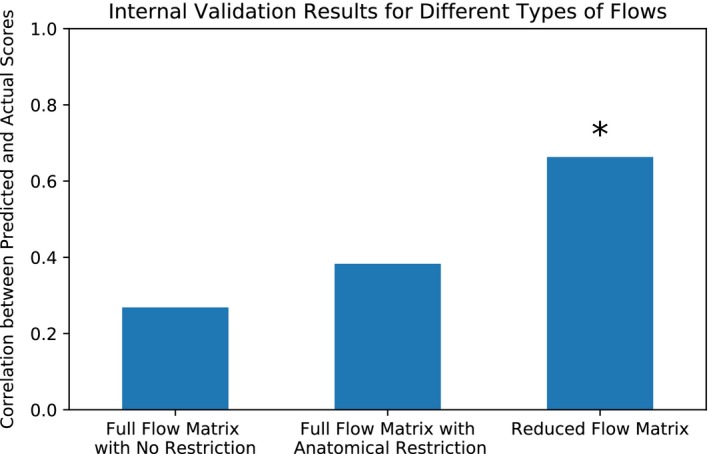
Results from three different types of flows: full flow matrix with no anatomical restriction (described in Methods), full flow matrix with anatomical restriction (described in Methods), and the reduced flow matrix (described in Methods). Here, we see that models based on the reduced information flow matrix significantly predict individual differences in attentional performance in the gradCPT sample, whereas the models based on the other two flow matrices did not yield significant predictions

We evaluated three different versions of the information flow matrices: information flow matrix with no macroscale group restriction, full information flow matrix with macroscale group restriction (see Figure 2), and reduced information flow matrices (see Figure 2). Figure 5 displays these results. Introducing anatomical restriction via macroscale grouping when calculating the maximum flow improves the predictive power of information flow‐based predictive models. Furthermore, the model with the reduced dimensionality (from 268 × 268 node × node to 20 × 20 lobe × lobe) was the most significant at predicting behavior.

**Figure 6:**
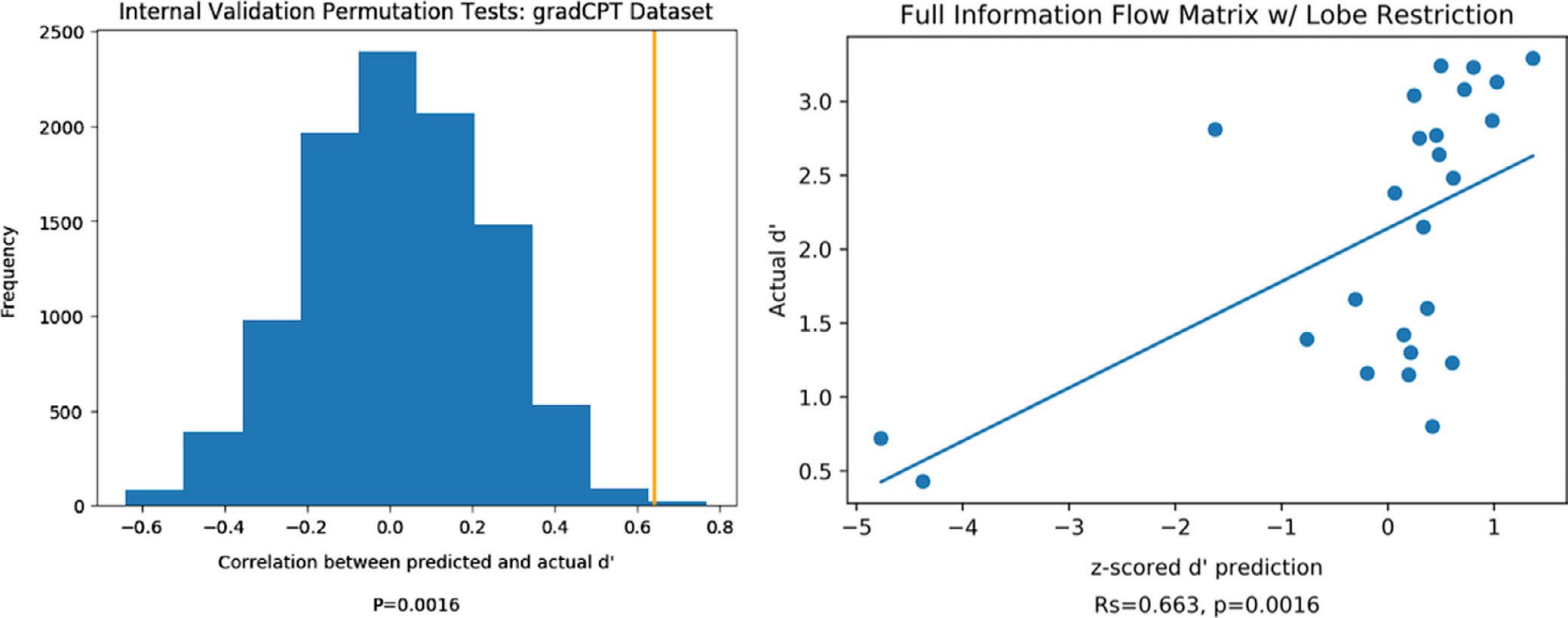
Internal validation results. Predicted and actual *d′* results were correlated using Spearman's rank correlation. Statistical significance was determined by randomly permuting subjects’ *d′* scores for 10,000 iterations, repeating the prediction analysis, and determining the fraction of correlations between predicted and actual scores that were as extreme as the original data. The relationship between observed and predicted *d′* scores remains significant in permutation testing if the two lowest predicted *d′* scores are excluded (*ρ *= 0.568, *p *= 0.0048)

Figure 6 displays the results for the reduced information flow matrices (the rightmost model in Figure 5). The model's predictions strongly and significantly correlated with observed behavioral scores (*ρ* = 0.663, *p *= 0.0002). The number of PCA components was determined through a nested LOOCV loop within the training set (see section “Model validation: Internal dataset”). The selected number of PCA components used differs in each iteration in the LOOCV loop since the training set of each iteration slightly differs. Out of each of the 25 iterations, the median selected number of components was 12, with a standard deviation of 4.14.

### 3.3 External validation results

**Figure 7:**
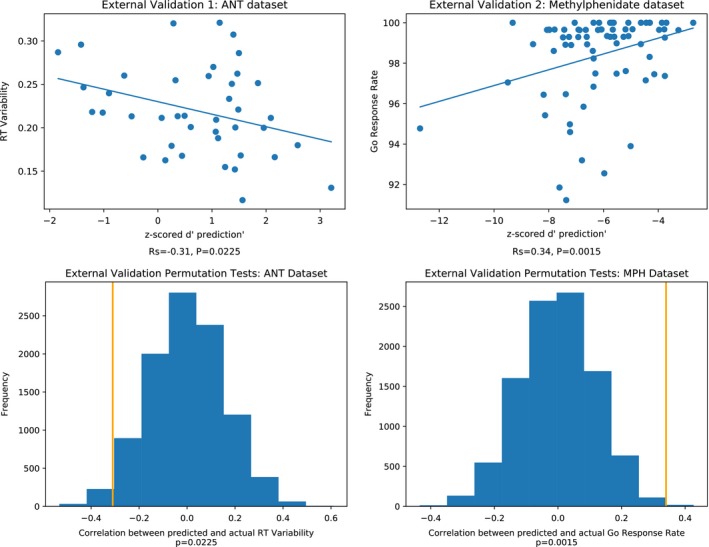
External validation results for Attention Network Task (ANT) and methylphenidate (MPH). Both ANT and MPH predictions were statistically significant. All relationships between observed and predicted behavioral scores are in the expected direction, as gradCPT *d′* and stop‐signal go rate scores correspond to better attention but higher ANT RT variability scores correspond to worse attention

We applied the model that performed the best in internal validation (reduced information flow) to the external validation datasets. Just as in internal validation, model performance was evaluated using Spearman's rank correlation on predicted and actual scores and statistical significance was determined using a 10,000‐iteration permutation test. The number of PCA components was determined through a LOOCV loop within the training set (see section “Model validation: External datasets”). The selected number of components in the external validation model was 12, which was the same as the median selected number of components in the internal validation model. The results across the three external datasets are shown in Figure 7.

The ANT and stop‐signal task datasets’ predictions were statistically significant (ANT: *ρ* = −0.31, *p*=0.0225; stop‐signal task: *ρ* = 0.34, *p *= 0.0015). Note that the ANT correlation coefficient is negative, because our CPM model was trained to predict gradCPT performance, so higher predicted scores correspond to better sustained attention. RT variability is negatively associated with sustained attention, so we expect model predictions to be negatively correlated with ANT performance scores.

### 3.4 Distribution of predictive flows

**Figure 8:**
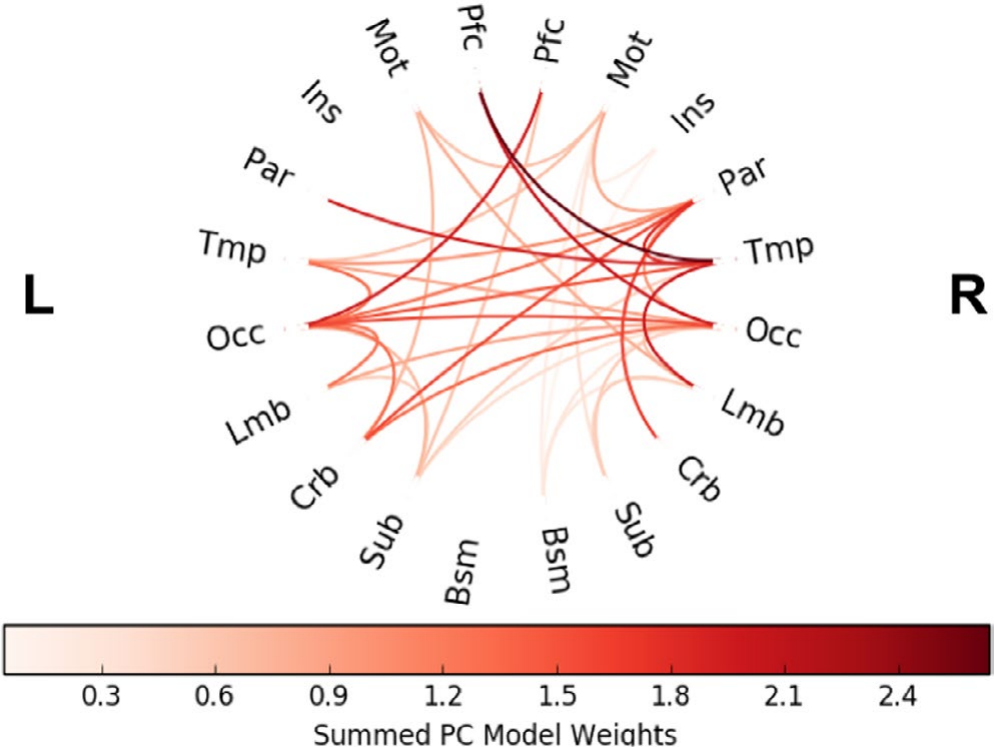
Distribution of flows included in the final predictive model in external testing. The connections are colored by their relative contribution to the model. A flow's contribution was calculated by summing the absolute value of its weight in a principal component across all components

Figure 8 shows the distribution of flows that were included in the predictions in external validation colored by their contribution to the linear model. The final model used for external validation tuned the number of principal components to 12, which was found by optimizing the LOOCV predictions within the training set. In Figure 7, a flow's contribution was calculated by summing the absolute value of its weight in a principal component across all 12 components. The connections that contributed the most to the principal components were between the right temporal lobe and left prefrontal cortex, from the right temporal lobe to the left cerebellum, and from the left prefrontal cortex to the left occipital lobe. Out of all 400 flows in the information flow matrix, 20 of those flows (one for each of the 20 regions) measure information flow within a certain region (“within flows”). The only within flow that was found to be predictive of behavior was the flow within the left occipital lobe.

### 3.5 Comparison with previous results

**Figure 9:**
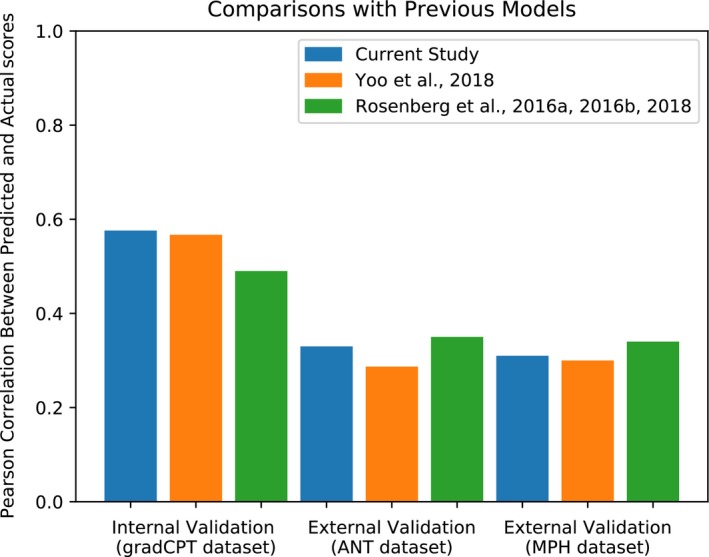
Comparison of our results with those of previous studies. We compared our model to other models that, like our model, were trained on task‐based fMRI and applied to resting‐state fMRI data. Yoo et al. (2018) compare different models, so just as we used the model that performed best in internal validation, and we compared the model in Yoo et al. (2018) that performed best in internal validation when trained on task‐based fMRI and applied to resting‐state fMRI. Note that we previously evaluated predictions based on Spearman's correlation. However, since previous publications were evaluated based on Pearson's correlation, all the results reported here are with Pearson's correlation

Figure 9 compares the current study's results with previously published predictions on the same datasets. We compared our model with other studies that trained models on task‐based fMRI data and applied models to novel participants’ resting‐state fMRI. Results demonstrate that predictions based on information flow perform comparatively to predictions that were based on more traditional measures of functional connectivity. Although information flow does not perform strictly better than measures used in the previous studies, it is important to highlight that information flow in addition theoretical capability of capturing nonlinear relationships. Therefore, due to its theoretical advantages in nonlinear analyses and network structure analysis and its success in predicting behavior, information flow may be a potentially useful measure of functional connectivity.

## 4 DISCUSSION

In this study, we proposed a new measure of functional connectivity “information flow” that abstracts the brain as a flow network of bits and quantifies functional connectivity as the amount of bits flowing between regions. We validate this proposed measure by using a machine‐learning framework to build a model that predicts individual differences in behavior. Specifically, we utilized an approach similar to that of Yoo et al. (2018) of building a connectome‐based predictive model (Shen et al., 2017) to predict individual attention scores across three independent datasets. Our CPM was able to use information flow to predict attention scores of novel individuals in three datasets (gradCPT, ANT, and SST) from resting‐state fMRI data. These results show a proof of principle that demonstrates that information flow can help characterize functional brain organization relevant to behavior.

Information flow accomplishes nonlinear analysis of signals via the use of TE. Previous research has shown that nonlinear analysis of fMRI signals can reveal results that linear analysis cannot. For example, Su et al. (2013) found that functional connectivity calculated from nonlinear methods differentiated schizophrenic patients from healthy patients with higher accuracy than that of linear methods. They also found that the strength of certain nonlinear functional connections increased in patients with schizophrenia, whereas their linear counterparts did not. Thus, it is important that information flow is able to predict behavior as well as Pearson's correlation, because it will have the added theoretical advantage of being able to elucidate nonlinear interactions that linear methods would not be able to. Here, we provide a proof of concept that information flow predicts individual differences in attention. Future work may explore whether nonlinear interactions captured by information flow offer benefits for predicting other behaviors and cognitive abilities across a variety of contexts.

Information flow abstracts the brain as a flow network of nodes that send bits of information to each other. To quantify how much information is flowing between particular regions, we used maximum flow. In internal validation, we tested three different types of information flow: the full (node by node) information flow with no anatomical restriction, the full (node by node) information flow with anatomical restriction, and reduced (lobe by lobe) information flow (see Figure 5). We saw that reduced information flow was the most significant at predicting behavior.

In general, the macroscale grouping used for maximum flow plays an important role in calculating information flow. We used the lobe groupings, which capture the gross anatomy of the brain and facilitate interpretability. We used the lobe grouping rather than a grouping focused on brain function, such as the functional networks described in Finn et al. (2015), because we wanted to introduce groupings that would provide an anatomical constraint to the paths of information flow. We saw that providing such an anatomical constraint helped the predictive models’ performance (Figure 5). We also used the lobe groupings to reduce the flow matrix (Figure 2f), because we observed in internal validation that having a smaller feature space helps predictive models (Figure 5). There are other types of groupings, some based on anatomy and some based on function, that may better capture macroscale brain organization and improve behavioral predictions. Testing other macroscale groups is a useful future direction. It is also possible to calculate information flow without restrictions as we have done in this study (see Figure 5). However, the calculation of information flow without restrictions (i.e., without applying a macroscale grouping) is computationally intensive given that, for each flow calculation, edges spanning the entire whole‐brain graph are taken into account. We showed here that one can use macroscale groups to reduce the computation time and, in some cases, obtain numerically better prediction results. Whether or not to use macroscale groups and impose restrictions on the amount of edges used in the calculation of each flow in order to reduce the computation time depends on the user of the method and their specific data.

We use CPM (Finn et al., 2015; Rosenberg, Finn, et al., 2016a; Shen et al., 2017) in order to predict behavior from functional connectivity measured by information flow. Unlike other studies that use CPM, we used PCA to aggregate features before inserting them it into a linear model (see Figure 3), whereas other studies take the sum of the features (Rosenberg, Finn, et al., 2016a) or use partial least squares regression (Yoo et al., 2018). We used PCA, instead of the feature aggregation methods used in previous studies, because we saw that it had numerically the highest performance in internal validation (see Figure S1).

It is certainly possible to use other flow constructs to abstract the brain as a flow network of bits. For example, the minimum cost flow problem involves finding the cheapest way of sending a certain amount of flow through a flow network (Ahuja et al., 2014). Rather than modeling the brain as a flow network designed to maximize the number of bits transported across regions, it is possible that we can instead model the brain as a flow network designed to flow a certain number of bits as efficiently as possible. Depending on one's specific data, it may be worth considering modeling information flow using different solutions than used for the maximum flow problem.It is also important to reiterate that when we discuss the transfer of bits of information via TE, we refer to *predictive* information transfer, which is distinct from *causal* information transfer (Lizier & Prokopenko, 2010). Predictive information transfer between brain regions is a statistical concept that does not imply that a physical connection causes measured information transfer. Although we cannot use TE to infer causal effects or the existence of physical processes between different brain regions, predictive information transfer can be useful in predicting behavior. Lizier & Prokopenko (2010) describe predictive information transfer and causal information flow (or causal effect) as two useful, but distinct, concepts. In a complex system, causal information flow is a microlevel property that can study causal relationships within the details of the system and can only be determined by intervention in the system. In contrast, predictive information transfer is a macrolevel property that can study the emergent computation of the system. In its microlevel viewpoint, causal effects are not effective in studying the emergent computation of the system, because intervening in a system to study certain variables’ causal relationships blocks the influence of other variables that are relevant to the emergent computation. When we use information flow in this study, we are using predictive information transfer to predict the emergent computation of the brain, which is behavior.

We used maximum flow to consider the topological structure underlying the nodes when estimating their functional connectivity. However, using maximum flow has some disadvantages. First, the algorithm needs capacities for each edge: the maximum amount that can flow directly within that edge. We used pairwise transfer entropies between nodes as those capacities, but we cannot infer the true capacity of direct information transfer. Therefore, each TE value can only serve as a lower bound on the amount of information transfer possible between those nodes. Additionally, when we use maximum flow from node *A* to node *B*, we are only describing the maximal amount of information that could flow from node *A* to node *B*. Therefore, in a situation such as that depicted in Figure 1, the information flowing from *A* to *B* may be independent of the information from *B* to *C*. In other words, our measure of information flow does not measure the exact amount of information flow between regions, but measures a lower bound on the maximal amount of information flow between regions. It is also important to emphasize that we are not claiming that information flow is necessarily better than more traditional measures to functional connectivity such as the Pearson correlation. Instead, we are presenting information flow as a new measure that has capabilities of measuring nonlinear interactions, has graph‐theoretic analysis, and can be successful in predicting behavior from brain data. Despite the limitations, the current results demonstrate that comparing the lower bound of the maximal amount of information flow across people can help us predict individual differences in behavior.

## CONFLICT OF INTERESTS

The authors declare no competing financial interests.

## Data Availability

The data that support the findings of this study are from Rosenberg et al. (2016a, 2016b) and Rosenberg et al. (2018). Restrictions may apply to the availability of these data. Requests for data should be directed to the corresponding author(s) of Rosenberg et al. (2016a, 2016b) and Rosenberg et al. (2018).
