# Supplementary figures and images for: An information network flow approach for measuring functional connectivity and predicting behavior
Source: Brain Behav. 2019 Jul 9;9(8):e01346. doi: 10.1002/brb3.1346 (PMC6710195; doi:10.1002/brb3.1346)

Internal Validation Results for Different Types of Feature Aggregation Methods

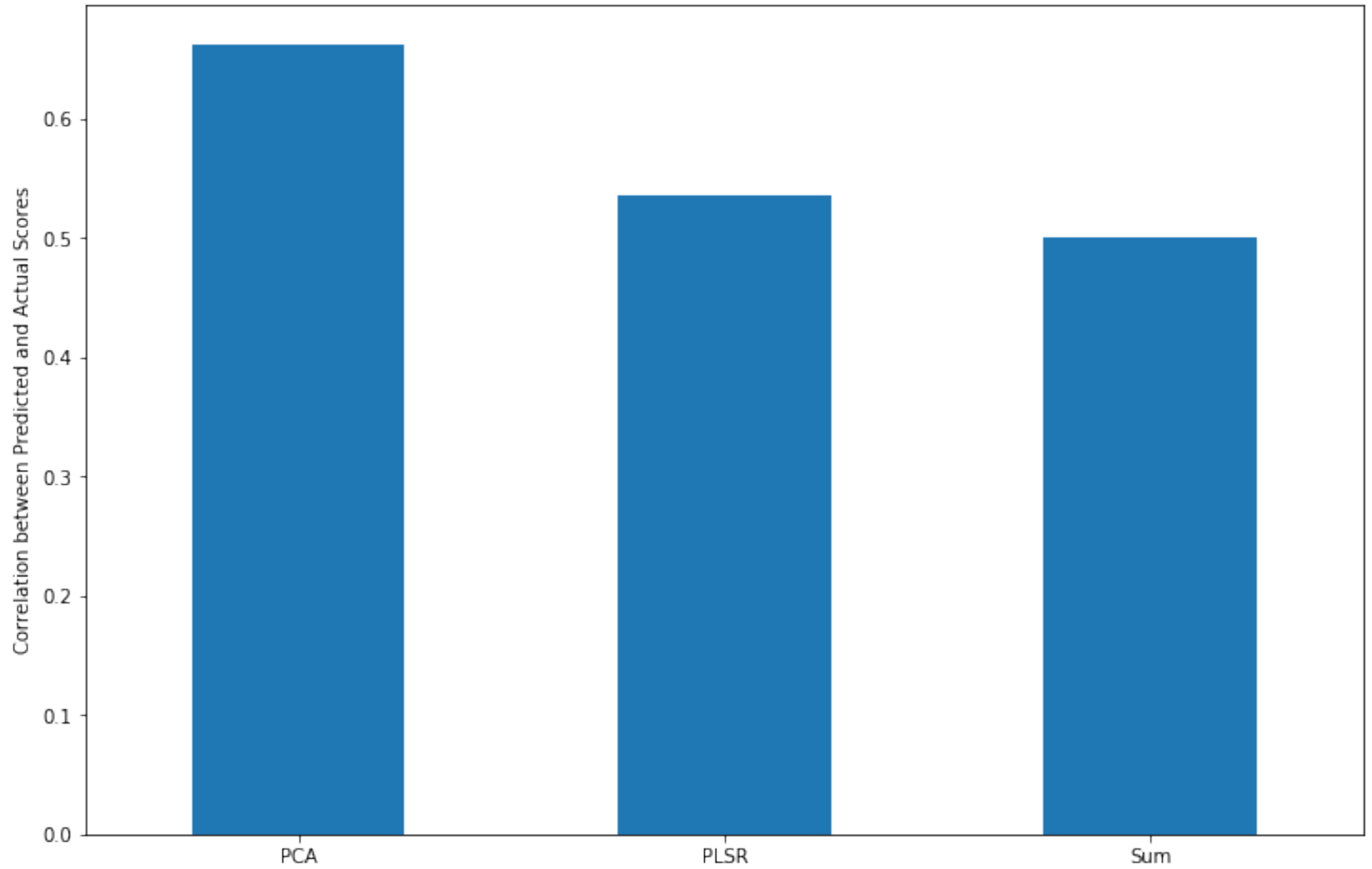

Supplement: Supplementary file 1 [file BRB3-9-e01346-s001.pdf]

Drop in Internal Validation Performance After Computational Lesioning

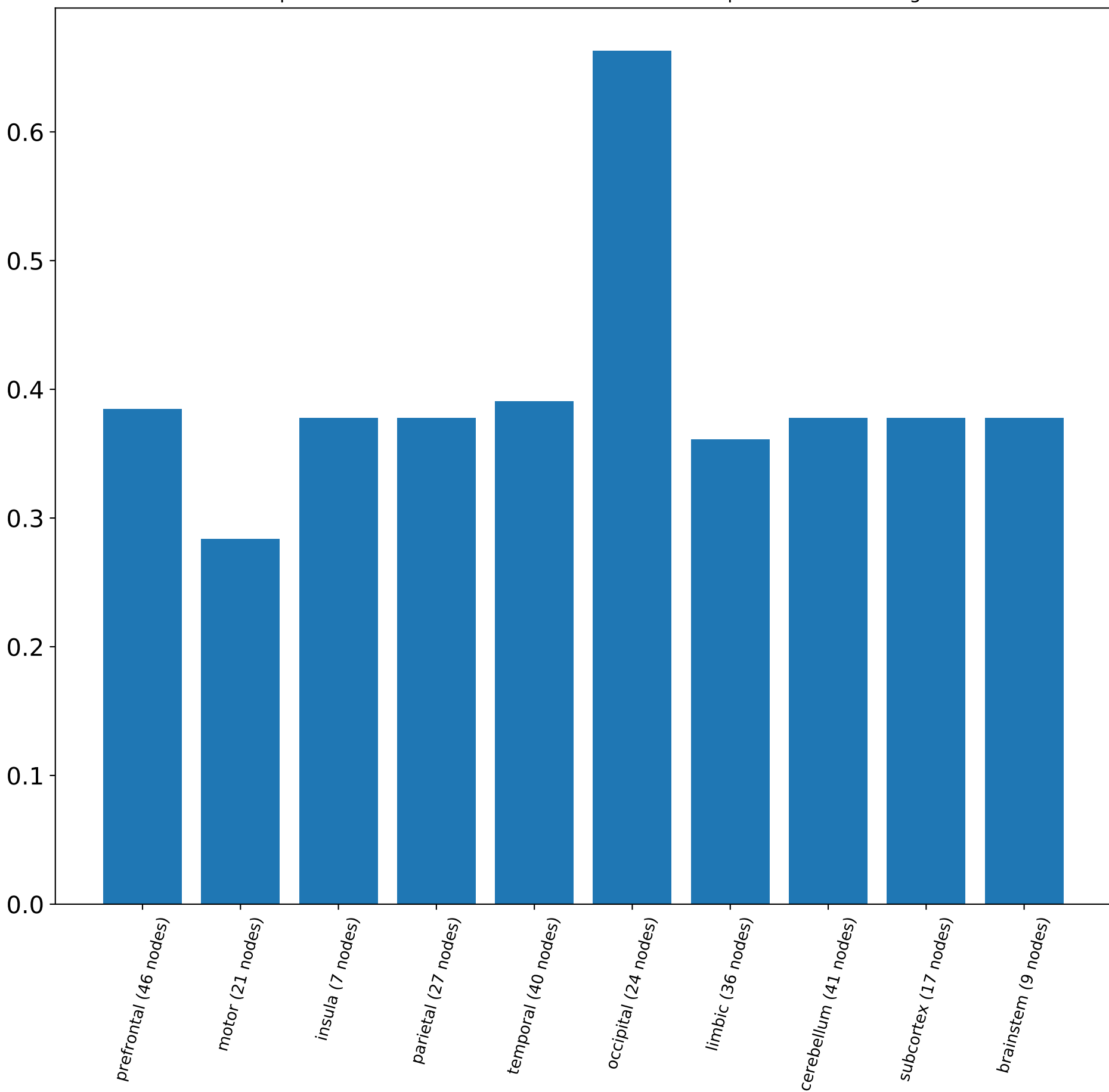

Supplement: Supplementary file 3 [file BRB3-9-e01346-s003.pdf]
