# Supplementary material for: An information network flow approach for measuring functional connectivity and predicting behavior
Source: Brain Behav. 2019 Jul 9;9(8):e01346. doi: 10.1002/brb3.1346 (PMC6710195; doi:10.1002/brb3.1346)
Supplement: Supplementary file 2 [file BRB3-9-e01346-s002.pdf]

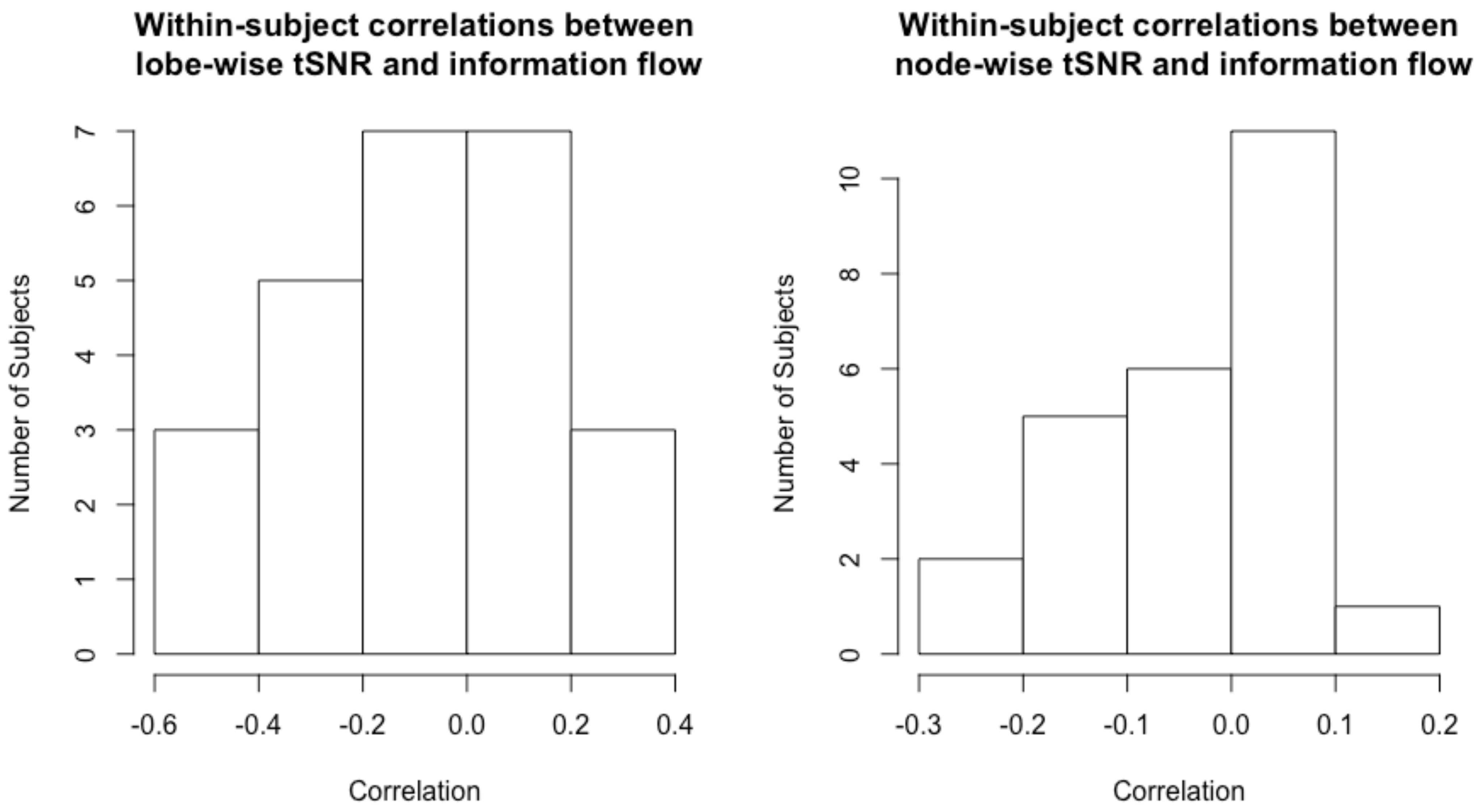
Within-subject correlations between lobe-wise tSNR and information flow
Number of Subjects
0
1
2
3
4
5
6
7
-0.6
-0.4
-0.2
0.0
0.2
0.4
Correlation
Within-subject correlations between node-wise tSNR and information flow
Number of Subjects
0
2
4
6
8
10
-0.3
-0.2
-0.1
0.0
0.1
0.2
Correlation
